# Microencapsulation of antioxidant phenolics from tamarind seed peels using chia gum and maltodextrin

**DOI:** 10.1038/s41598-025-89792-6

**Published:** 2025-02-25

**Authors:** Azza M. Abdel-Aty, Abdul Aziz M. Gad, Amal Z. Barakat, Saleh A. Mohamed

**Affiliations:** Molecular Biology Department, National Research Centre, Dokki, Cairo, Egypt

**Keywords:** *Tamarindus indica*, Peels, Encapsulation, Phenolic compounds, Stability, Bioavailability, Biochemistry, Biological techniques, Biotechnology

## Abstract

*Tamarindus indica* seeds/seed peels (TSP) are the main waste products from the tamarind industry and contain valuable bioactive antioxidant-phenolic compounds that promote human health; however, their application is limited due to their instability and poor solubility. Encapsulation is becoming more important in several industries due to its potential for preserving and delivering valuable and delicate bioactive compounds. This study targets the microencapsulation of TSP-phenolic compounds for incorporation into functional food formulations. By employing the freeze-drying method, three microcapsule formulations were developed using chia gum (CG), maltodextrin (M), and a mixture of the two (M/CG) as coating materials. The formed M-, M/CG-, and CG-microcapsules showed remarkable encapsulation efficiency of 88.0, 90.0, and 95.0%, respectively. They preserved most of the TSP-phenolic content (87.5–96.3%) and antioxidant activity (86.0-98.8%). They demonstrated higher digestibility percentages in the intestinal media (53.0–70.0%) than in the gastric media (29.0–36.0%), especially the microcapsules coated with CG. They kept the TSP-antioxidant-phenolic content safe at 40 °C for 2 months. The microcapsules demonstrated improved microstructures, swelling, solubility, and moisture content. Crosslinking and enhanced thermal stability were also proven for microcapsules via FTIR and thermogravimetric studies. In addition, the prepared microcapsules displayed better antimicrobial activity against the examined bacterial strains, with minimum bactericidal concentrations ranging from 0.61 to 1.4 mg/mL. In conclusion, the encapsulation improved the stability, bioavailability, and antibacterial properties of TSP-phenolic compounds, making them suitable for food and pharmaceutical applications.

## Introduction

Plant-derived polyphenolic compounds are one of the greatest groups of natural compounds with various valuable biological actions and have multiple positive health impacts on humans, such as antioxidant, anticancer, antimicrobial, anti-inflammatory, antidiabetic, and anti-snake properties^1–7^. These bioactive properties strongly depend on the bioavailability of phenolic compounds in the human body. The delivery structure and form of phenolic compounds into the human body significantly influence their bio-accessibility and, consequently, their bioavailability. The polyphenolic compounds interact with different macromolecules during digestion, including proteins, lipids, carbohydrates, and fibers, significantly impacting their bioavailability in the human body^8,9^. These interactions, including hydrogen bonding and hydrophobic binding, influence enzyme activities, amino acids, lipid oxidation, and carbohydrate digestion. The binding with fibers also impacts the release and bioavailability of polyphenols, affecting their overall stability and absorption in the human body. These interactions determine how effectively polyphenols are absorbed and their subsequent health benefits^10^. Moreover, various food processing techniques can either enhance or diminish the nutritional value and bioavailability of bioactive polyphenolic compounds. Some phenolic compounds showed reduced bio-accessibility/bioavailability in the digestive system due to their poor stability in aqueous solutions, poor stability under gastric intestinal tract (GIT) conditions, and difficulty absorbing through membranes^11^.

Encapsulation is a process in which the active compounds (like phenolic compounds) are coated with a safe biopolymer (coating material) that protects them from negative external impacts and allows the controlled release of these active compounds in a specific environment^12^. Additionally, encapsulation can mask unwanted flavors and tastes of the phenolic compounds and improve their health benefits, shelf life, stability, and bioavailability in GIT^13^. Coating materials are important in encapsulation since they affect active controlled release, target delivery, and bio-accessibility. They should have distinctive properties such as safety, flexibility, strength, stability, impermeability, and biodegradability^14,15^.

Maltodextrin (M), a modified starch, is a widely used carbohydrate-based encapsulant known for its thermal stability, neutral taste, and easy digestibility. Its low viscosity and ability to enhance the solubility of hydrophobic substances contribute to its effectiveness in forming stable microcapsules. Additionally, M is highly compatible with various bioactive compounds, making it a trusted choice for encapsulating active phenolic compounds in the food and pharmaceutical sectors^16^. Chia gum (CG), a polysaccharide complex derived from chia seeds, is recognized for its impressive viscosity, emulsification, gelation properties, and excellent thermal stability. CG is particularly effective in creating strong, flexible films that protect bioactive compounds, promoting moisture retention and controlled release. Furthermore, CG is biodegradable and non-toxic, making it ideal for sustainable applications across different industries^17^. CG and M offer superior stability and bioavailability compared to gelatin, alginate, and starch. Unlike gelatin and alginate, they are more stable, easier to handle, and cost-effective for large-scale, applications^18,19^. Due to these advantages, CG and M were selected to optimize the encapsulation efficiency, stability, and controlled release of the bioactive phenolic compounds tested.

*Tamarindus indica* L. (commonly named tamarind, family Fabaceae) is a tropical evergreen tree native to Africa and Southern Asia. It is widely grown for its fruits, used in several food products, and in many concoctions as medicinal treatments^20,21^. Recently, studies on tamarinds have focused mainly on its seeds. Tamarind seeds and seed peels are widely generated as agro-industrial by-products during tamarind pulp processing. These materials are often discarded or underutilized, leading to significant waste accumulation. Despite this, they represent a valuable yet largely untapped resource due to their rich composition of polyphenols, antioxidants, and other bioactive compounds. These properties position tamarind seeds and peels as promising candidates for sustainable value-added applications^22^. They are a rich source of antioxidant phenolic compounds such as dihydroxybenzoate, dihydroxy-phenyl acetate, procyanidins, and epicatechin, with potent antioxidant, antimicrobial, and anticancer activities^23,24^. The tamarind seed water extract was encapsulated using wax-incorporated emulsion gel beads^25^. The tamarind seed peels (TSP) also contain flavonoids, phenolics, and tannins, with potential antioxidant activity^26^. TSP extracts have several bioactive properties, including antimicrobial, anti-diabetic, anti-inflammatory, lipid peroxidation reduction, anti-tyrosinase, and anti-hyperlipidemic activities^27,28^. Therefore, TSP-phenolic compounds have the potential to provide low-cost nutritional and therapeutic value, making them good components in functional food formulations. However, the encapsulation of TSP-phenolic compounds to conserve their therapeutic and nutritional properties has not been reported. Thus, this work aims to produce new microencapsulates/formulations of tamarind seed peel phenolic-rich extract (TSPE) using the wall materials maltodextrin (M) and purified chia gum (CG) (mixed and non-mixed) to protect its nutritional and therapeutic properties. In addition, the produced microcapsules were evaluated for encapsulation efficiency, storage stability, gastrointestinal bioavailability, micromorphology, functional group composition, thermal stability, and antibacterial properties. *Escherichia coli* and *Staphylococcus aureus* were selected for this study due to their relevance as major human pathogens; *E. coli* is responsible for foodborne illnesses and *S. aureus* commonly causes resistant infections. TSP, known for its antimicrobial properties^26^, will be tested against these bacteria to evaluate their broad-spectrum antimicrobial potential, particularly against Gram-negative and Gram-positive strains.

## Materials and methods

### Materials

Tamarind (*Tamarindus indica*) (sour cultivar) seeds and peels were collected from fruits purchased from local markets in Egypt. Maltodextrin DE 8–15, Folin-Ciocalteu reagent, 1,1-diphenyl-2-picrylhydrazyl (DPPH), (2,2′-azino-bis-3-ethylbenzothiazoline-6-sulfonic acid) (ABTS), gallic acid, Trolox, pepsin, and pancreatin were obtained from Sigma-Aldrich Co. All materials and experiments were carried out according to the relevant institutional, national, and international guidelines and legislation.

### Extract preparation

The dried tamarind seed peels (10 g) were homogenized in 100 mL for two times of methanol/water (80:20, v/v) and shaken in a water bath shaker at 40 °C for 12 h. The mixture was centrifuged at 5000 rpm for 10 min and filtered using Whatman paper (No. 1). The solvent was then evaporated using a rotary evaporator (Thermo Fisher Scientific, USA), and the concentrated extract was frozen at -80 ºC for 24 h, followed by lyophilization in a freeze dryer (Benchmark Freeze Dryer, SP Scientific (VirTis), USA). The dry extract was stored at 4 °C until further use^29^.

### Chia gum preparation

Chia seeds were immersed in distilled water at a 1:20 (w/v) ratio for 2 h at 45 ºC. The resulting mucilage was separated by filtration. One part mucilage to three parts ethanol was mixed and the resulting precipitate was then collected, dried, and ground^29^.

### Encapsulation of TSPE

Maltodextrin DE 8–15 (M) and chia gum (CG) were applied as coating materials to encapsulate the dried tamarind seed peel extract (TSPE). 100 mg of M or CG was individually dissolved in 100 mL of distilled water with 500 rpm stirring and at 40 ºC. Coating material solutions were prepared an hour before the encapsulation process. At three different formulas, the TSPE and coating material solutions were combined using the following ratios M: TSPE (5:1), M + CG: TSPE (2.5 + 2.5:1), and CG: TSPE (5:1). Each mixture/formula was stirred at 1000 rpm for 1 h until obtaining a good dispersion. For freeze-drying, each prepared mixture/formula was frozen at -80 ºC and then placed into a freeze-dryer chamber at -60 ºC under pressure of 0.05 bar and kept under these conditions for 24 h. The proper encapsulation conditions were selected based on preliminary experiments and insights from some previous studies^29,30^.

### Total polyphenolic and antioxidant activity

Twenty mg of TSPE or the microcapsules were combined with 2 mL of ethanol, acetic acid, and water solution at a 50:8:42 ratio for 2 min. Each mixture was vortexed for 2 min and filtered using a 0.5 µm filter^31^. To determine the total polyphenolic content (TPC), each sample (100 µl) was mixed with 100 µl of Folin-Ciocalteu reagent and 800 µl of distilled water, and the mixture was then incubated for five minutes at room temperature. Each mixture was combined with 500 µl of 20% sodium carbonate solution and incubated for 30 min, and the absorbance at 750 nm (Shimadzu UV-2401PC Spectrophotometer, Japan) was then measured^32^. Gallic acid equivalent (GAE) was used as a standard to express the TPC. To determine the antioxidant activity using DPPH, each sample (100 µl) was mixed with 900 µl of 0.1 mM DPPH solution and incubated for 15 min in the dark at room temperature, and the absorbance at 517 nm was then measured^33^. To determine the antioxidant activity using ABTS, each sample (100 µl) was mixed with 900 µl of ABTS solution and incubated for 1 min at room temperature, and absorbance was measured at 734 nm^34^.

The antioxidant activity was measured using Equation 1:

1 $${\text{DPPH or ABTS scavenging }}\% \, = \, \left( {{\text{OD}}_{{{\text{Control}}}} {-}{\text{OD}}_{{{\text{Sample}}}} } \right)/{\text{OD}}_{{{\text{Control}}}} \times { 1}00$$

Trolox equivalent (TE) was used as a standard to express the total antioxidant activity (TAA).

### Surface polyphenolic content (SPC) and encapsulation efficiency (EE) of the prepared microcapsules

Twenty mg of each microcapsule was added to 2 mL of a methanol and ethanol solution at a 1:1 ratio for 2 min. Each mixture was filtered after two minutes under a vortex^35^. The Folin-Ciocalteu reagent was used to measure each SPC microcapsule filtrate as previously mentioned. Equation 2 was used to determine the EE of the prepared microcapsules:

2 $${\text{EE }}\% \, = \, \left( {{\text{TPC}} - {\text{SPC}}/{\text{TPC}}} \right) \, \times { 1}00$$

### Simulated gastrointestinal digestion

Two conditions were employed to determine the TPC released from synthesized microcapsules: Simulated Gastric Fluid (SGF) and Simulated Intestinal Fluid (SIF). Ten mL of SGF, containing 0.2 mM NaCl, 4 mg pepsin, and 0.1 M HCl at pH 2.0, was added to each microcapsule, and it was shaken for 2h at 100 rpm and 37 ºC. After gastric digestion, part of the mixture was kept at − 20 ºC, and the entire mixture was allowed to reach a pH of 7.3 before adding the SIF solution (4 mg pancreatin) and shaking it for 2h at 37 ºC at 100 rpm^36^. After each stage of digestion, each sample was neutralized using a 100 mM NaHCO_3_ solution, centrifuged for 10 min at 5000 rpm, and the released phenolic content was measured using the Folin-Ciocalteu method. The digestibility % was determined using Equation 3:

3 $${\text{Digestibility }}\% \, = \, \left( {{\text{PC}}_{{{\text{Release}}}} /{\text{TPC}}} \right) \, \times { 1}00$$

PC _Release_: Phenolic content released from the microcapsule, TPC: Total phenolic content found in microcapsule

### Shelf-life stability

The developed microcapsules and TSPE powder were kept in separate brown vials and stored at 40 ºC for two months. As mentioned in Total polyphenolic and antioxidant activity section, the samples’ total polyphenolic and antioxidant activity retention was examined at weeks 0, 2, 4, 6, and 8 during storage^29^.

### Physical properties of the prepared microcapsules

#### Moisture

Every microcapsule (100 mg) was dried for 24 h at 105 °C, and the variation in weights (mg) of each microcapsule before and after drying was examined as the moisture (%)^37^.

#### Solubility

Every microcapsule (100 mg) was dissolved in 10 mL of deionized water, vortexed for a minute, kept at 37 °C for 30 min, and centrifuged for five minutes at 5,000 rpm. Every supernatant was dried for 24 h at 105 °C. The solubility (%) was determined by measuring the difference between the initial and the supernatant weights^37^.

#### Swelling

Each 100 mg microcapsule was immersed in distilled water at 28 °C for 3 h. After vacuum-filtering the samples and using filter paper to remove any extra water, the weight was once again measured^38^. The pre- and post-swelling weight differences (mg) for each microcapsule were calculated as the swelling percentage (%).

### Surface morphology

To analyze the microstructure of the coating materials (M and CG-gum) and the developed microcapsules, a scanning electron microscope (SEM) (FE-SEM, Quanta FEG 250) was operated at an accelerating voltage of 20 kV^29^.

### FTIR-spectra

The obtained microcapsules and the coating materials (M and CG-gum) were subjected to FTIR examination applying a Bruker ALPHA-FT-IR-Spectrometer. The 400–4000 cm^1^ scanning wave range was applied^29^.

### Thermal-properties

The thermal properties of the prepared microcapsules and their coating materials were examined using thermogravimetric (TGA) and differential thermogravimetric (DTG) analyses. Sample runs were carried out between 40 and 800 °C at a steady heating rate of 10 °C/min^39^.

### Antibacterial activity

The antibacterial properties of both TSPE and the obtained microcapsules were examined against *Escherichia coli* O157-H7 ATCC 51659 (gram-negative bacterial strain) and *Staphylococcus aureus* ATCC 13565 (gram-positive bacterial strain) using dilution and colony counting processes^39^. Each investigated bacterial strain was cultivated in Falcon tubes containing Mueller–Hinton broth supplemented with varying concentrations of the tested samples. The cultures were incubated with shaking at 37 °C for 17 ± 1.0 h. Following incubation, the Falcon solutions were cultured onto Mueller–Hinton agar and incubated under the same conditions (37 °C for 17 ± 1.0 h) to allow bacterial colonies to grow. Colony counts were performed after this incubation period. The antibacterial activity was evaluated by determining the minimum bactericidal concentration (MBC), defined as the lowest concentration required to eliminate 99.9% or more of the initial bacterial inoculum.

### Statistical analysis

The statistical evaluation was conducted using one-way ANOVA, followed by Tukey’s post-hoc analysis, utilizing GraphPad Prism 5 software. All values are expressed as the mean ± SD (n = 4), with statistical significance determined at P < 0.01.

## Results and discussion

### Total phenolic content (TPC) and total antioxidant activity (TAA)

TSPE contains various phenolic compounds that exhibit antioxidant and potential medicinal properties. Prior studies have identified several key phenolic compounds in tamarind seed peels, including gallic acid, catechin, tannins, ellagic acid, quercetin, chlorogenic Acid, and ferulic acid. These phenolic compounds contribute to the antioxidant activity and potential health-promoting properties of tamarind, highlighting its relevance for pharmacological and nutraceutical applications^40^.

The TPC of the produced M-, M/CG- and CG-microcapsules was marginally lower (35.0, 37.5, and 38.5 mg GAE, respectively) than that of the non-encapsulated TSPE (40.0 mg GAE) as displayed in Table 1. This reduction in TPC post-encapsulation is likely attributed to the freeze-drying process. While freeze-drying is effective in preserving bioactive compounds, it may still lead to oxidative degradation and structural changes in phenolics due to prolonged exposure to low temperatures and vacuum conditions. Furthermore, mechanical stress from water removal during the process can disrupt the encapsulating matrix, potentially exposing phenolic compounds to degradation or reducing their extractability. This result was observed in previous reports that encapsulated polyphenolic compounds via the freeze-drying technique^29,30,41,42^. In addition, the TAA of the produced M-, M/CG- and CG-microcapsules was slightly reduced (27.5, 29.1, and 30.7 µmol TE, respectively, using DPPH) and (45.2, 48.4, and 50.6 µmol TE, respectively, using ABTS) compared to the initial TAA of the TSPE (32.0 µmol TE, using DPPH) (51.2 µmol TE using ABTS) with activity retention percentages of 86, 91, 96%, and 88.2, 94.5, 98.8, respectively (Table 1). This little reduction in TAA of the prepared capsules may be due to the decrease in TPC compared to the original TSPE extract.

**Table 1:** Total phenolic content (TPC), surface phenolic content (SPC), encapsulation efficiency (EE), and total antioxidant activity (TAA) of Tamarind seed peel extract (TSPE) encapsulated in different ratios of Maltodextrin (M) and Chia gum (CG) using the freeze-drying technique.

Sample	Wall: TSPE ratio	TPC mg GAE	SPC mg GAE	EE (%)	TAA µmol TE using DPPH	TAA retention % Using DPPH	TAA µmol TE using ABTS	TAA retention % using ABTS
TSPE	–	40.0 ± 2.2^a^	–	–	32.0 ± 2.2^a^	100.0^a^	51.2 ± 2.5^a^	100.0^a^
M	5.0:1	35.0 ± 2.0^b^	4.2 ± 0.21^a^	88.0^a^	27.5 ± 2.0^b^	86.0^b^	45.2 ± 2.1^b^	88.2^b^
M + CG	2.5 + 2.5:1	37.5 ± 2.1^a^	3.7 ± 0.17^b^	90.0^b^	29.1 ± 2.3^a^	91.0^c^	48.4 ± 2.3^a^	94.5^a^
CG	5.0:1	38.5 ± 1.7^a^	1.9 ± 0.10^c^	95.0^c^	30.7 ± 2.0^a^	96.0^d^	50.6 ± 2.0^a^	98.8^a^

Values are presented as means ± SD (n = 4); results in the same column with different superscripts (a, b, c) are significantly different at (*p* < 0.01).

The results also reveal that the highest levels of antioxidant-phenolic compounds were preserved when CG was used as the wall material compared with M to encapsulate the TSPE. M is a polysaccharide polymer, while CG is a hetero-polysaccharide polymer with a minor protein content besides the sugar content. This small amount of protein in the CG structure gives hydrophobic properties besides the hydrophilic properties of its sugar content. Both hydrophobic and hydrophilic properties of chia gum are responsible for the good emulsifying properties of chia gum^17,29,43^. This may explain the greater retention of the antioxidant-phenolic content in CG- and M/CG-capsules than in M-capsules.

### Encapsulation efficiency (EE)

The EE analysis is a vital tool for evaluating the success of the encapsulation process. This analysis can demonstrate the protection offered to the TSPE phenolic compounds and their functional properties. The EE% values of the produced M-, M/CG-, and CG-microcapsules are 88.0, 90.0, and 95.0%, respectively (Table 1). The generated CG microcapsule showed higher phenolic content, antioxidant activity, and EE % than other M and M/CG microcapsules. This finding indicates that the phenolic compound encapsulating process depends critically on the selected coating material. CG has excellent film-forming ability, creating strong and flexible capsules that protect core materials effectively. It also possesses good emulsifying and gelling properties, allowing it to form a stable three-dimensional network that safeguards core compounds, especially TSPE-phenolic compounds, by maintaining their stability and activity. In contrast, M has moderate film-forming ability, poor emulsifying properties, lacks gelling capacity, and often requires additives like gum and stabilizers to improve its performance^17,30^. Reduced EE values (27.0– 75.0%) were previously observed for maltodextrin-microcapsules of blueberry, cactus pear juice, black carrot, black carrot juice, pomegranate juice, spent coffee grounds, and blueberry juice^31,44–47^. Consistent with our findings, the Arabic gum and maltodextrin combination demonstrated great EE% values (93.0 to 98.3%) when employed to capsulate various phenolic compounds^42^. Garden cress gum microcapsule also recorded an efficient EE value of 98.2% among eight generated microcapsules, with EE values (80.3 to 96.5%)^30^.

### Simulated gastro-intestinal digestion

**Fig. 1:**
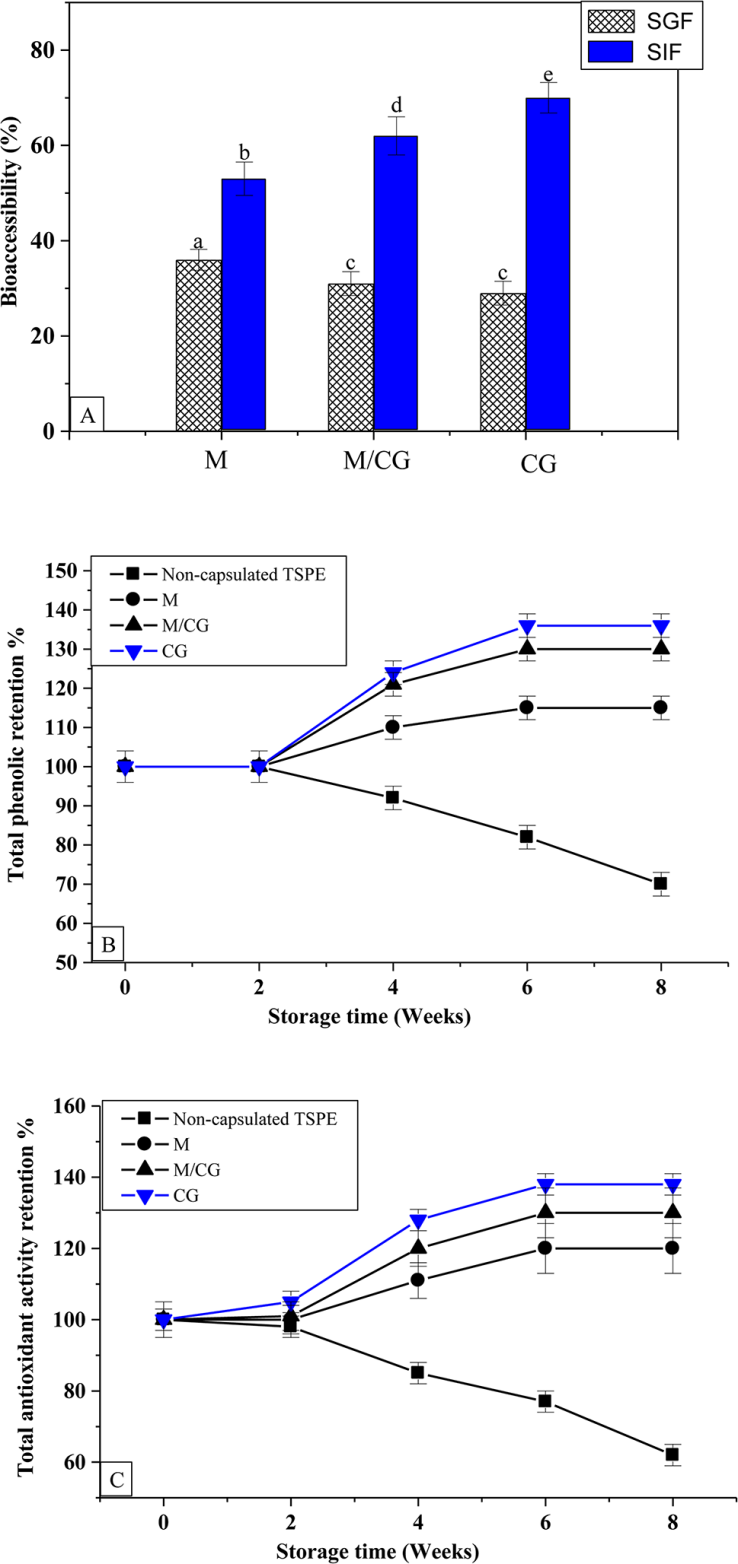
(**A**) In vitro release profile of phenolic content from the prepared microcapsules in simulated gastric fluid (SGF) and simulated intestine fluid (SIF), (**B**, **C**) Storage stability of the total phenolic content and total antioxidant activity of the TSPE and the prepared microcapsules stored at 40 °C for 8 weeks. TSPE: Tamarind seed peel extract M: Maltodextrin-microcapsule; M/CG: Maltodextrin-Chia gum microcapsule and CG: Chia gum microcapsule. Values are provided as means ± SD (n = 4). Different letters in the same column are statistically different at (*P* < 0.01).

Generally, the TPC of most plant extracts decreases during digestion, likely due to oxidation and polymerization with less soluble, high-molecular-weight compounds. Additionally, TPC is sensitive to gastrointestinal pH, leading to stability variations that affect its bioavailability^48^. Encapsulation technology was used to protect bioactive compounds, enhance their stability, and extend their shelf life^49^. Herein, the TSPE-phenolic compounds released from the generated microcapsules were examined in two pH conditions (SGF and SIF) to monitor TSPE-phenolic compound delivery at an absorption site. The digestibility % of the generated microcapsules in SGF and SIF after 2 h at 37 °C is highlighted in Fig. 1A. In SGF media, the TSPE-phenolic compounds release (digestibility %) of the M-, M/GC-, and CG-microcapsules recorded 36.0, 31.0, and 29.0%, respectively. While, in SIF media, the TSPE-phenolic compound release (digestibility %) of the M-, M/GC-, and CG-microcapsules were markedly elevated and recorded at 53.0, 63.0, and 70.0%, respectively. All the generated microcapsules showed higher digestibility values in the SIF compared to the SGF, especially the microcapsules coated with chia gum. Microcapsules coated with CG showed greater stability in SGF, likely due to the acid-resistant properties of the gums^50^. However, the higher digestibility values in SIF suggest that the CG coating gradually breaks down or swells in the intestinal environment, facilitating the controlled release of the encapsulated bioactive^17,29^. This finding indicates that chia gum enhances the digestion of TSPE-phenolic compounds in the intestinal media and offers more protection in the stomach medium. CG appears to be an appropriate covering for the TSPE-phenolic compounds, as evidenced by the decreased release of phenolic compounds under gastric conditions and increased them in intestinal conditions more than maltodextrin coating material. Therefore, the produced M/CG and CG microcapsules can be added to several food preparations. Similar findings were previously documented for the phenolic compounds released from microcapsules containing moringa extract coated by maltodextrin and pectin^51^, microcapsules coated by maltodextrin and Arabic gum^42^, microcapsules containing chia sprout extract coated by chia gum^29^ and microcapsules containing garden cress sprout extract coated by garden cress gum^30^.

### Stability during storage condition

Figure 1B,C show changes in the TPC and TAA of the developed microcapsules compared to non-capsulated TSPE kept at 40 ºC for eight weeks. The TPC and TAA levels of the TSPE extract gradually decreased to 70 and 62%, respectively, after 8 weeks of incubation under 40 °C. This result showed that TPC and TAA in TSPE are easily available and unprotected when interacting with the environment. Remarkably, the TPC and TAA levels of the developed M, M/CG, and CG capsules elevated by 115, 130, 136, and 120, 130, 138%, respectively, after 8 weeks of incubation under 40 °C. The increase in TPC during storage may be due to the hydrolysis of phenolic glycosides of TSPE, accelerated by higher temperatures. This process releases free phenolic aglycones, which are more detectable and reactive to the Folin-Ciocalteu reagent and DPPH free radicals. Encapsulation likely helped preserve the phenolic compounds, with the TPC increase primarily attributed to glycoside hydrolysis and temperature-induced changes^52^. Additionally, the wall materials of the prepared microcapsules safeguarded these free phenolic compounds. Likewise, during 50 days of incubation at 60 °C, the TPC of the purple cactus pear extract rose in all three microencapsulates by almost twice^52^. The TPC and TAA levels of chia sprout extract highly increased in microcapsules coated by chia gum after 60 days of incubation at 40 °C^29^. The TPC retention stayed stable in microcapsules developed for banana-peel extract coated by maltodextrin and Arabic gum, during 4 weeks of incubation under 40 °C^37,53^. However, the cherry juice microencapsulated by maltodextrin and Arabic gum retained 90% of its TPC after 4 weeks of incubation under 38 °C^54^. Based on the findings, the properties of the coating materials including the core retention ability and embedding capacity, have a major impact on the capsule storage stability. So, the prepared M, M/CG, and CG microcapsules could keep the TSPE-antioxidant-phenolic compounds safe for a long time, even in harsh storage conditions.

### Physical properties of microcapsules

#### Moisture content

Moisture concentration has a major impact on viscosity, flow state, and storage stability in various food systems. The food industry aims for moisture concentrations in the range of 1.0–7.0% to maintain the stability of powder products during storage^55^. The produced CG- M/CG- and M-microcapsule moisture contents (4.1, 5.2, and 8.0%, respectively) (Table 2) fell within or close to this range, and all obtained results were close to those reported in several studies, such as the moisture contents of the garden cress gum GG and S/GG microencapsulates containing garden cress sprout extract (6.3 and 4.6%, respectively)^30^ and chia gum and gelatin CG/G- and CG microcapsules containing chia sprout extract (4.0 and 7.0%, respectively)^29^. The moisture contents of the Arabic gum and gelatin microcapsules containing black raspberry extract ranged from 1.97 to 6.69%^56^.

**Table 2:** Moisture, solubility, and swelling properties of the obtained M, M/CG, and CG microcapsules.

Microcapsule	Moisture (%)	Solubility (%)	Swelling (%)
M	8.0 ± 0.30^a^	80 ± 4.0^a^	105 ± 0.2^a^
M/CG	5.2 ± 0.12^b^	50 ± 2.1^b^	197 ± 11^b^
CG	4.1 ± 0.24^c^	25 ± 1.1^c^	207 ± 21^c^

M: Maltodextrin-microcapsule; M/CG: Maltodextrin-Chia gum microcapsule and CG: Chia gum microcapsule. Values are provided as means ± SD (n = 4). Different letters (a, b, c) in the same column are statistically different at (*P* < 0.01).

### Solubility properties

The solubility values of the M/CG- and M-microcapsules were 50 and 80%, respectively, while the CG-microcapsule recorded a lower solubility value of 25% (Table 2). Reduced solubility delivers the core molecules into the target controllably, gradually, and slowly. Based on observations, the higher solubility of M-microcapsule may be due to the hydrophilic character of maltodextrin^57^. The combination of maltodextrin and chia gum reduced the solubility of the M/CG microcapsule. Likewise, garden cress sprouts extract microcapsules based-maltodextrin showed higher solubility (95 and 99%), than other microcapsules based on garden cress gum (47%)^30^.

### Swelling properties

The generated M, M/CG, and CG microcapsules showed swelling values of 105, 197 and 407%, respectively (Table 2). The better-swelling properties of both M/CG and CG microcapsules might be attributed to the gelling property of the chia gum, which swells when it absorbs water and forms three-dimensional network structures. This property can keep the structure and function of core content. This clarifies why the TSPE antioxidant-phenolic compounds are well-preserved in the M/CG and CG microcapsules. Garden cress sprouts extract-microcapsules based-garden cress gum showed superior swelling properties (141%) than others based on maltodextrin (102%)^30^.

### SEM-Micromorphology

**Fig. 2:**
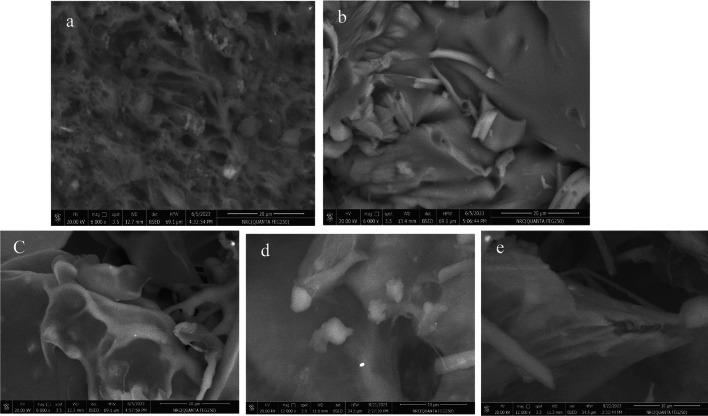
SEM images of the purified chia gum (**a**) and maltodextrin (**b**) as coating materials, M-microcapsule (**c**), CG-microcapsule (**d**), and M/CG-microcapsule (**e**). M: Maltodextrin-microcapsule; M/CG: Maltodextrin-Chia gum microcapsule and CG: Chia gum microcapsule.

Figure 2 shows SEM images of the coating materials (maltodextrin and chia gum) and the generated M, M/CG, and CG microcapsules. The surfaces of the two coating materials exhibited significant differences in morphology. The maltodextrin surface was smooth, whereas the chia gum surface was rough and highly irregular (Fig. 2a,b). Molecules with rough surfaces have the potential to reduce the release of core material by trapping it within their structures. The surfaces of the M-, M/CG-, and CG-microcapsules showed more regular forms and were smoother and more uniform (Fig. 2c,d,e). It was observed that the developed microcapsules served as stores where the coating material effectively trapped the core material. Such structures result in microcapsules with a larger storage capacity and enable the retention of more antioxidant-phenolic compounds. Therefore, the prepared microcapsules are a superior option for maintaining the antioxidants of TSPE and the encapsulated TSPE-antioxidant phenolic compounds can maintain their stability during storage.

### FTIR-spectra

**Fig. 3:**
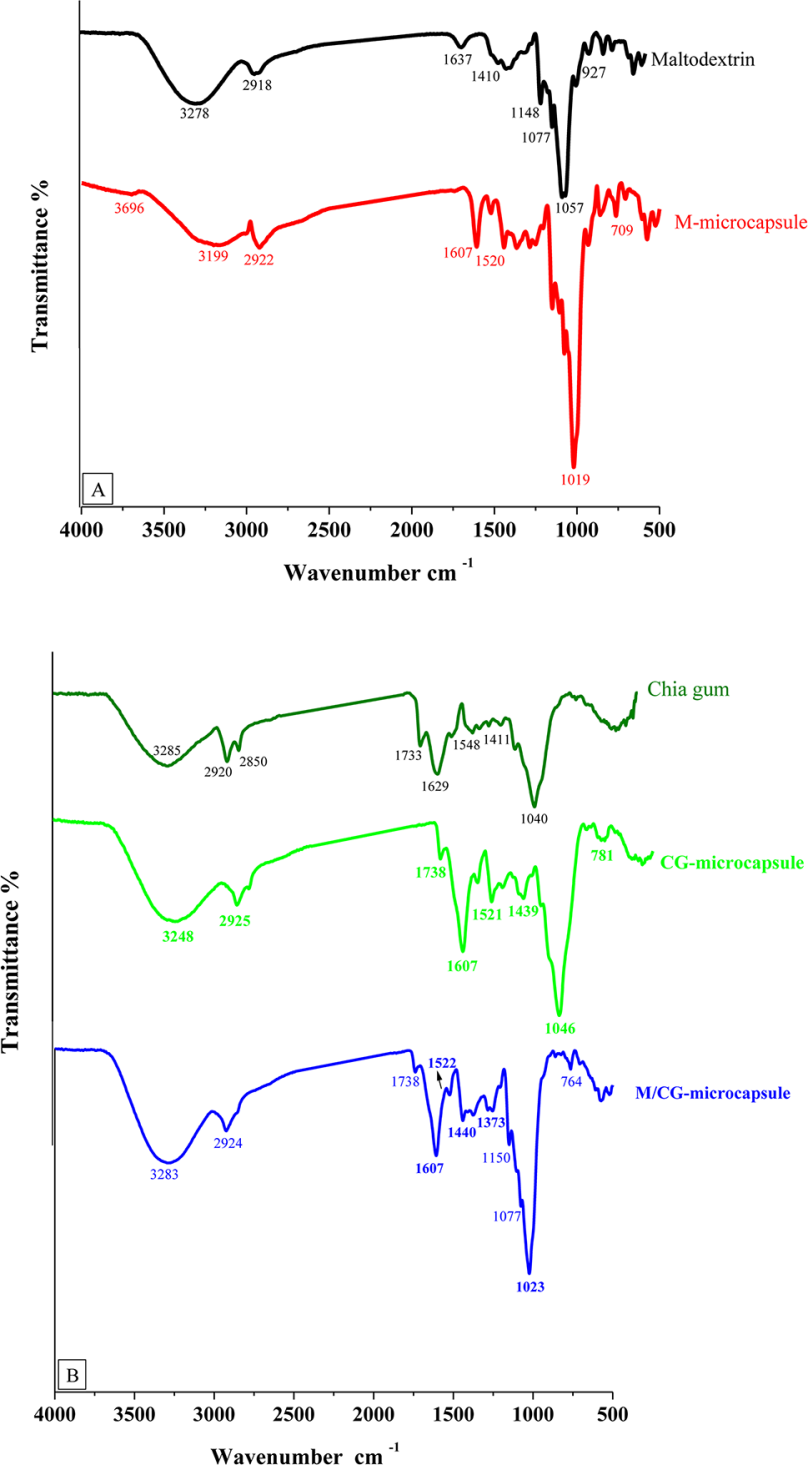
FTIR spectrum of the maltodextrin and M-microcapsule (**A**), the purified chia gum, CG-microcapsule, and M/CG-microcapsule (**B**). M: Maltodextrin-microcapsule; M/CG: Maltodextrin-Chia gum microcapsule and CG: Chia gum microcapsule.

Figure 3A,B show the IR spectrum of coating materials, M and CG, and the produced microcapsules (M-, CG- and M/CG-microcapsules). In the M-spectrum, a strong band of O–H-stretching at 3278 cm^−1^, the CH_2_–band at 2918 was created by the C-H stretching. Further significant bands for M can be found around 1057 and 927 cm^−1^, signifying the stretching vibrations of the C–O–H groups and C–O–C groups in the anhydro-glucose ring, respectively. All these peaks were typical for the M structure^58^. The prepared M-microcapsules spectrum showed vibrations of several O–H groups at 3199 cm^−1^ (intense broadband), and the peak at 2922 cm^−1^ was linked to the symmetric and asymmetric stretching of the C-H bond in methyl groups of polyphenolic compounds. In addition, the aromatic C–C stretching at 1520 cm^−1^, and CH_2_ rocking at 709 cm^−1^ were all associated with the structures of phenolic compounds^59^. Further, the 3278, 2918, 1637, and 1057 peaks were shifted to 3199, 2922, 1607, and 1019 cm^−1^ respectively, with great intensity as seen in Fig. 3A.

In the CG spectrum, the O-H stretch at 3258 cm^−1^, the C-H aromatic ring stretching, and the CH_3_-group at 2920 and 2850, respectively, –COO-groups at 1548 and 1411 cm^−1^ and the negative carbonyl (C–O) band at 1040 cm^−1^ were detected (Fig. 3B). These determined hydroxyl and carbonyl groups were typical for chia gum^17,29^. For the prepared CG microcapsules, a great intense broad band of vibrations for multiple O–H groups, C–C stretch at 1439 cm^−1^, and C–H_2_ at 781 cm^−1^, were all connected to phenolic compound structures^30^. Moreover, the O–H, C–H, –COO–, and C–O bands at 3285, 2920, 1548, 1411, and 1040 were changed and intensified at 3248, 2925, 1521, 1439, and 1046 cm^−1^, respectively, as seen in Fig. 3B.

For the prepared M/CG-microcapsules, a higher-intense band of O–H group vibrations at 3283 cm^−1^, C–C stretch at 1440 cm^−1^, and CH_2_ at 764 cm^−1^, were all also associated with phenolic compound structures^30^. Further, the bands at 3285, 2920, 1548, 1411, and 1040 were changed to 3283, 2924, 1522, 1440, and 1023 cm^−1^, respectively, with greater intensity (Fig. 3B). It can be observed that, in the three generated microcapsule spectra, the majority of functional groups of coating materials and TSPE-phenolic compounds were demonstrated with clear shifting, robust absorption, and wide intensities after the encapsulation process, especially, in CG microcapsules. This finding may be due to the formation of multiple intermolecular hydrogen bonds between TSPE phenolic compounds and coating polymers. Several carboxyl and hydroxyl groups in TSPE-phenolic compounds enhanced the chance for many hydrogen bonds to form between molecules^60^. These results indicate the interaction between the TSPE phenolic compounds and the functional groups of the coating materials. Following microencapsulation, the coating materials protected the most functional TSPE groups. CG contains bioactive phenolic acids with antioxidant properties^61^. The improved encapsulation of TSPE-phenolic compounds in CG-microcapsules can be attributed to strong molecular interactions, such as hydrogen bonding between the hydroxyl groups of TSPE phenolic compounds and the functional groups of CG. These interactions, supported by the bioactive phenolic acids in CG, likely reinforce the encapsulation matrix, resulting in greater stability and bioavailability of the encapsulated substances^60^.

### Thermal properties

**Fig. 4:**
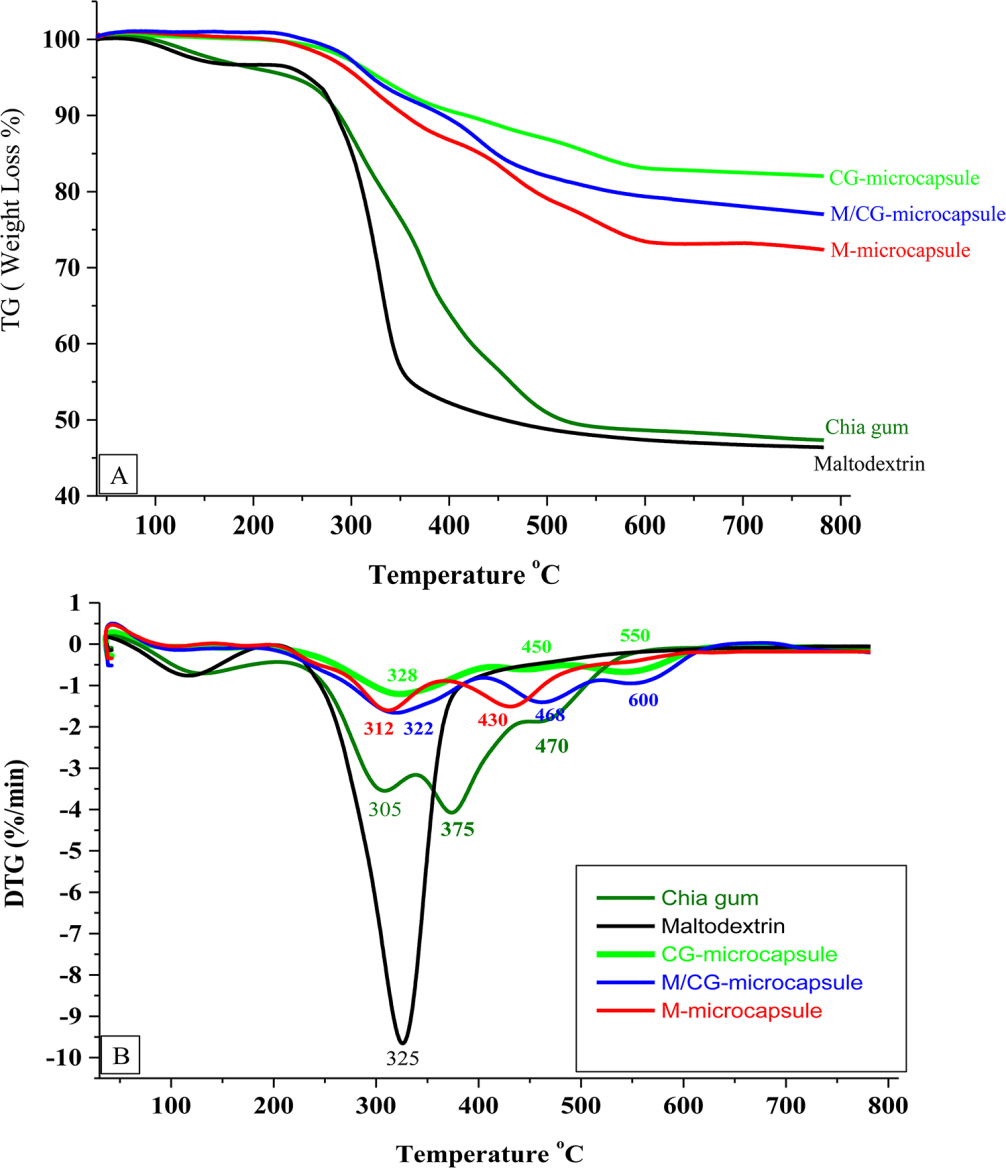
TGA curves (**A**) and DTG curves (**B**) of the purified chia gum and maltodextrin as coating materials and the obtained microcapsules. M: Maltodextrin-microcapsule; M/CG: Maltodextrin-Chia gum microcapsule and CG: Chia gum microcapsule.

The thermal properties of the three prepared microcapsules (CG, M/CG, and M) compared to their coating materials were examined using TG and DTG techniques (Fig. 4A,B). For TG thermal analysis, the first thermal decomposition of examined coating materials (CG and M) was determined between 135-170°C with a mass loss of 5%, while the initial degradation of the three produced microcapsules (CG, M/CG, and M) was started between 250 and 260 °C with a mass loss of 1.2%. The second mass degradation of the examined coating materials was determined between 270 and 370 °C with about ~50% mass loss. However, the three produced microcapsules (CG, M/CG, and M) retained 72–83% of their masses at 600 °C, as seen in Fig. 4A. In DTG analysis, the maximum degradation rate for maltodextrin was detected at 325 °C and for chia gum at 305 and 375 °C. However, the maximum degradation rate for the produced CG-, M/CG-, and M-microcapsules was detected at 328, 450, 550, and 322, 468, 600, and 312, 430°C, respectively (Fig. 4B). The produced microcapsules require three or two distinct temperatures to break down. The elevations in the first and final degradation temperatures of the prepared microcapsules compared to their covering materials demonstrate an improvement in the thermal stability of the prepared microcapsules following encapsulation. This improvement may be due to the forming of intra and intermolecular hydrogen bonds after the encapsulation process. Some studies confirmed the thermal stability improvement following the encapsulation such as the spent-coffee phenolic compounds microencapsulated with maltodextrin and Arabic gum^47^ and garden cress phenolic compounds microencapsulated with garden cress gum^30^.

The enhanced thermal stability of microencapsulated TSP-phenolic compounds, as demonstrated by TG and DTG techniques, is highly relevant for their application in high-temperature food processing and long-term storage. In processes such as pasteurization and sterilization, where food is exposed to elevated temperatures, encapsulation can protect phenolic compounds from thermal degradation, maintaining their antioxidant properties. Additionally, the encapsulation technique protects TSP-phenolic compounds during long-term storage, maintaining their stability, bioactivity, and nutritional benefits even under fluctuating temperatures^62^.

### Antibacterial properties

Antibacterial properties of the prepared microcapsules (CG-, M/CG-, and M) compared with unencapsulated TSPE were examined against *E. coli* and *S. aureus* bacterial strains. The prepared CG- and M/CG showed significantly greater (*P* < 0.01) antibacterial efficacy against the examined bacteria, with lower minimum bactericidal concentrations (MBC) (0.61-0.86 mg/mL) compared to M-microcapsules (1.1–1.4 mg/mL), TSPE (0.83–1.1 mg/mL) and Amoxicillin (1.3–2.2 mg/mL) as seen in Table 3. These results suggest that encapsulating the TSPE with CG enhanced its antibacterial activity. This enhanced antibacterial activity could be attributed to the potential antibacterial effects of CG on the tested bacterial strains. CG is rich in phenolic acids, including p-hydroxybenzoic acid, chlorogenic acid, caffeic acid, p-coumaric acid, and ferulic acid^61^. These compounds exhibit strong antioxidant and antimicrobial properties. They can penetrate the cytoplasmic membrane, increasing its permeability and leading to the leakage of essential bacterial cell components, such as proteins, nucleic acids, and inorganic ions^63^. In addition, it can be observed that the unencapsulated TSPE and M-microcapsules showed more potent antibacterial activity than the Amoxicillin as a positive control. Some phenolic compounds of TSPE have powerful antimicrobial properties and may release H_2_O_2_ damaging bacterial cell membranes and inhibiting bacterial growth^21,26,64^. Moreover, the micro sizes of the prepared capsules help speed up bacteria ingestion, increase bacteria-cell membrane damage, and interrupt protein synthesis^65^. Some capsules, such as CEO, CS/GTO, S/GG, CG, CG/G, and G reported better antibacterial properties after encapsulation^30,39,66^.

**Table 3:** Antibacterial properties of non-capsulated TSPE and the obtained microcapsules. Amoxicillin is a positive control.

Sample	Bacterial strains MBC (mg/ml)	
	*S. aureus*	*E. coli*
TSPE	1.1 ± 0.05^a^	0.83 ± 0.02^a^
M	1.4 ± 0.07^b^	1.1 ± 0.03^b^
M/CG	0.86 ± 0.02^c^	0.70 ± 0.02^c^
CG	0.72 ± 0.03^b^	0.61 ± 0.02^d^
Amoxicillin	1.3 ± 0.31^b^	2.2 ± 0.03^e^

MBC: Minimum bactericidal concentration; TSPE: Tamarind seed peel extract M: Maltodextrin-microcapsule; M/CG: Maltodextrin-Chia gum microcapsule and CG: Chia gum microcapsule. Values are provided as means ± SD (n = 4). Different letters (a, b, c) in the same column are statistically different at (*P* < 0.01).

Overall, this encapsulation technology and prepared TSPE microcapsules have diverse applications in functional foods and pharmaceuticals. In the food industry, they improve the stability, bioavailability, and antibacterial activity of TSPE bioactive compounds in products such as beverages, dairy items, and nutritional supplements. In pharmaceuticals, they offer controlled release and antibacterial properties for drug formulations. Additionally, TSPE microcapsules can be applied in cosmetics to deliver antioxidants and antimicrobial agents, demonstrating their potential for innovation across many formulations.

## Conclusion

Chia gum, maltodextrin, and their mixture successfully encapsulated the TSP-phenolic-rich extract, forming three microcapsule formulations (CG-, M/CG-, and M). These microcapsules exhibited high encapsulation efficiency, retained significant phenolic content and antioxidant activity, and demonstrated superior antibacterial properties. Notably, they released more TSP-phenolic compounds in simulated intestinal fluid than in gastric fluid, confirming the protective role of the coating materials during the gastric stage. In addition to their functional properties, the microcapsules showcased prolonged storage stability at 40 °C, remarkable morphological characteristics, and robust interactions between the coating materials and TSP-phenolic compound functional groups. These features demonstrate the efficacy of encapsulation in improving the stability, bioavailability, and antibacterial activity of TSP-phenolic compounds, making TSPE microcapsules highly suitable for applications in functional foods, pharmaceuticals, and cosmetics.

### Potential applications and future research directions

The encapsulation technique used in this study is not limited to TSP-phenolic compounds; it can be effectively applied to a wide range of bioactive compounds, including vitamins, probiotics, essential oils, and flavonoids. Future studies could focus on evaluating the scalability of this encapsulation method for industrial applications and investigating the efficacy of these microcapsules in real food and pharmaceutical systems. Additionally, examining the long-term bioavailability and in vivo impacts of TSP microcapsules could provide valuable insights into their real-world applications.

## Data Availability

The datasets produced and/or examined in this study can be obtained from the corresponding author upon a reasonable request.
